# Agreement and diagnostic accuracy of noncycloplegic retinoscopy, subjective and autorefraction in a young adult Ghanaian population

**DOI:** 10.4314/gmj.v60i1.5

**Published:** 2026-03

**Authors:** Samuel Kyei, Jehu Azure, Randy Asiamah, Michael A Kwarteng

**Affiliations:** 1 Department of Ophthalmic Science, School of Optometry and Vision Sciences, University of Cape Coast, Cape Coast, Ghana; 2 Biomedical and Clinical Research, College of Health and Allied Sciences, University of Cape Coast, Ghana; 3 School of Optometry and Vision Sciences, University of Cape Coast, Cape Coast, Ghana; 4 Department of Clinical Optometry, School of Optometry and Vision Sciences, University of Cape Coast, Cape Coast, Ghana; 5 Optometry Unit, Department of Clinical Surgical Sciences, Faculty of Medical Sciences, The University of the West Indies, Saint Augustine Campus, Trinidad and Tobago

**Keywords:** Specificity, Sensitivity, Retinoscopy, Autorefraction, Subjective refraction, Ghana

## Abstract

**Objective:**

To determine the agreement and diagnostic accuracy of retinoscopy, subjective and autorefraction among young adults in a sampled Ghanaian population.

**Design:**

A descriptive cross-sectional design

**Setting:**

Optometry Department of Bishop Ackom Memorial Christian Eye Centre, Cape Coast, Ghana

**Participants:**

Refractive error data were obtained from 100 adults aged 18-35 years (200 eyes)using autorefraction, retinoscopy and subjective refraction

**Main outcome measures:**

Spherical equivalent, J_0_ and J_45_ values, and sensitivity and specificity for myopia and hyperopia detection.

**Results:**

The mean age was 23.38 ± 4.39 years, 61% females. This study used Bland–Altman analysis and intraclass correlation to compare refractive techniques. For spherical equivalents, retinoscopy and subjective refraction yielded high agreement (ICC=0.9), and autorefraction and subjective refraction exhibited significant agreement (ICC=0.8, p < 0.001). Retinoscopy and subjective refraction showed a significant correlation (ICC = 0.81, p < 0.001). Similarly, autorefraction and subjective refraction for J_0_ correlated (ICC=0.67, p < 0.001), and retinoscopy and subjective refraction showed low agreement on the J_45_ axis (ICC=0.01, p=0.48). Autorefraction and subjective refraction were weakly but significantly correlated (ICC=0.22, p < 0.001). The specificity and sensitivity of retinoscopy in detecting myopia are similar to those of autorefraction. The accuracy of retinoscopy was greater than that of autorefraction in detecting myopia. However, retinoscopy and autorefraction demonstrated equal accuracy in detecting hyperopia.

**Conclusion:**

In young adults, retinoscopy shows strong agreement with subjective refraction, demonstrating its consistency in evaluating spherical equivalents and the J_0_ axis. Although efficient, autorefraction showed reduced precision and less consistency for J_45_ axis determination.

**Funding:**

None declared

## Introduction

Uncorrected refractive error (URE) still ranks as the second most common cause of visual impairment worldwide, affecting individuals across all age groups.^1^ It is particularly detrimental in children, impacting learning and participation in educational settings.^2^ Among school-aged learners in Ghana, the prevalence of refractive error varies by region, from 1.7% to 22.6%.^3^,^4^

Adults with uncorrected refractive errors also face substantial challenges, as impaired vision affects quality of life, productivity, and financial independence.^1^ The prevalence of URE in adults varies globally, with high rates of myopia in Asia and lower rates in Africa.^1^ The economic impact of uncorrected refractive error is significant, with an estimated global productivity loss of USD 411 billion, underscoring the need for accessible and efficient vision correction methods.^5^

Subjective refraction is the benchmark for assessing refractive error in clinical practice.^6^ This method may pose difficulties, including limited comprehension of instructions, inconsistent responses, reduced fixation ability, and communication barriers in specific populations, such as young children, persons with cognitive disabilities, or those who exhibit inconsistent replies.^7^ Objective techniques such as retinoscopy and autorefraction offer faster and potentially more reliable alternatives. While retinoscopy provides flexibility and detailed information on optical aberrations, it requires technical skill and can yield variable results across examiners due to differences in proficiency and experience, among other factors.^6^–^8^ Autorefraction, while easier to administer, is often not accurate enough to replace subjective refraction entirely, attributable to accommodation influence, patient fixation management, inability to assess visual comfort, and variability in complex cases, such as irregular corneas and cataracts, among others.^8^

Despite advancements in objective refraction techniques^8^, there has been limited research to investigate the diagnostic accuracy of objective refraction techniques (retinoscopy and autorefraction), especially in adults in Africa. Inaccurate objective measurements may influence prescribing decisions, emphasising the need for structured clinical training and skill assessment. Globally, numerous studies have compared refractive characteristics between children and adults via these techniques.^9^–^17^ The studies in children generally show poorer agreement between objective methods (autorefraction, retinoscopy) and subjective refraction, largely because of active accommodation, whereas adults, especially young adults, show much closer agreement between objective and subjective measures, though astigmatic components (especially oblique J45) remain more variable across ages.^9^–^17^ However, in Africa, research has predominantly focused on children aged^18^, whereas other studies have focused on the best predictor of astigmatism.^19^ Consequently, there is a lack of data validating the effectiveness of objective techniques in adults on the continent. This gap creates uncertainty when extrapolating findings from paediatric studies to adults, whose refractive profiles and accommodative behaviour differ significantly. To address this, the present study evaluates the diagnostic accuracy and agreement of noncycloplegic retinoscopy and autorefraction relative to subjective refraction in young adults.

Establishing the reliability of these techniques in adults is essential for informing clinical decision-making, especially in settings where subjective refraction may be difficult to perform.

## Methods

This study used a descriptive cross-sectional design to collect refractive error data from various adult participants at a single point in time, under the same clinical conditions. By measuring all techniques during one visit, the design allowed direct comparison of their outputs without the influence of temporal changes in refractive status or accommodation. The target population included adults aged 18 to 35 years with refractive errors at the Optometry Department of Bishop Ackom Memorial Christian Eye Centre, Cape Coast, Ghana, between 1st February, 2024 and 31st July, 2025. The age range was chosen because evidence indicates a decline in accommodation amplitude (AOA) with age, thereby ensuring consistent testing parameters across participants. A purposive sampling technique was employed to select individuals with refractive errors. Patients visiting the study location were invited to participate, and those who met the criteria were randomly selected. Sample size was estimated in G*Power (version 3.1.9.7) for a correlation test under a bivariate normal model (two-tailed, α=0.05, power=0.95). Assuming an expected correlation of 0.80 between methods and a null correlation of 0.60, the required total sample size was 82. To accommodate the use of two eyes per participant and the intra-participant correlation, we inflated this to a target of approximately 90 participants (≈180 eyes), and further rounded to 100 participants (200 eyes) to ensure adequate precision across secondary analyses and allow for potential attrition.

The participants included adults aged 18 to 35 years with refractive errors who provided consent and completed all the examination protocols. The exclusion criteria were adults with known ocular pathologies that could affect vision (e.g., irregular corneas, cataracts), presbyopes, individuals who declined consent, and individuals with vision impairment from causes other than refractive error. Visual impairment was classified as distance visual acuity worse than 6/12 according to the World Health Organisation classification.

### Data collection and procedure

All testing was completed in a single visit under uniform clinical conditions to ensure comparability across techniques. An experienced optometrist conducted every assessment, thereby minimising interobserver variability. The anterior and posterior segments of each eye were screened for abnormalities using a penlight and direct ophthalmoscopy. The examiner inspected the lids, lashes, conjunctiva, cornea, anterior chamber, iris, lens clarity, vitreous, retina, and optic nerve head, documenting any signs of pathology or media opacity that could affect visual acuity or refractive measurement. Findings that might influence refraction, such as corneal scars, significant cataract, or retinal lesions, were recorded, and, when necessary, the participant was excluded from refractive testing or referred for further care.

Retinoscopy was performed at a working distance of 67 centimetres using a streak retinoscope. Each participant wore a trial frame fitted with a +1.50-diopter fogging lens to relax accommodation while the examiner swept the retinoscope beam across the pupil and observed the reflex. The examiner introduced spherical and cylindrical lenses into the trial frame to neutralise the reflex, adjusting lens power until a neutral or reversed reflex was obtained; plus cylinders were transposed to minus cylinder form when required for consistency with the clinic's refraction notation. The examiner noted the endpoint for sphere, cylinder power, and axis, and recorded the working-distance correction so the final retinoscopy result could be adjusted to the spectacle plane.

Autorefraction was performed with the closed-field Humphrey ZEISS autorefractor to provide an independent measure of refractive state. Participants were seated with their eyes aligned with the instrument's optical axis; the examiner adjusted the table height and seating position so the participant's pupils were level with the instrument, and verified correct placement of the chinrest and forehead rest to minimise head movement. Participants were repeatedly instructed to fixate on the distant target inside the instrument and to keep both eyes open and the head steady; multiple readings were taken when necessary and averaged, or the most consistent reading was selected. Any large discrepancies between autorefraction and retinoscopy were noted for further investigation during subjective refraction. Care was taken to ensure proper alignment by adjusting the autorefractor table height and the participant's seating position to keep the participant's eyes level with the instrument's optical axis. The examiner also verified the placement of the chinrest and forehead rest for stability and instructed participants to maintain a steady head position throughout the measurement. Subjective refraction was performed at a distance of 6-meters using a Snellen chart to establish the starting acuity and the working endpoint. The participant wore a trial frame with the previously determined fogging lens to control accommodation while the examiner presented a sequence of spherical lenses to refine the spherical equivalent, followed by cylindrical lenses to correct astigmatism. A Jackson Cross Cylinder was used to refine both the power and axis of astigmatic correction through repeated comparison questions, and the examiner adjusted lenses according to the participant's responses until the clearest, most comfortable letters were identified. Once monocular endpoints were established, binocular balancing techniques were applied to equalise the perceived clarity between the two eyes and to ensure comfortable binocular vision; final adjustments were made until the participant achieved a binocular visual acuity of 6/6 or better.

Throughout testing, the examiner maintained consistent lighting, standardised chart luminance, and a quiet testing environment to reduce variability. All instrument settings, working distances, lens powers, transpositions, and participant responses were recorded in the participant's chart, along with any ocular findings from the penlight and ophthalmoscopic examinations. If any measurement suggested an ocular abnormality or if the participant could not reliably complete the procedures, the examiner documented the issue and arranged appropriate follow-up or referral.

### Study Definitions

Refractive errors were categorised according to the criteria outlined in Table 1, consistent with a prior study^20^. Refractive error classification was based on the results of noncycloplegic subjective refraction.

**Table 1:** Classification of refractive errors

Refractive error	SE (Diopters)	Sphere (Sph) (Diopters)	Cylinder (Cyl) (Diopters)*
**Emmetropia**	−0.50 to +0.50		|Cyl| ≤ 0.75
**Myopia^†^**	SE ≤ −0.50	Sph ≤ 0	
**Hyperopia^‡^**	SE ≥ +0.50	Sph ≥ 0	|Cyl| ≤ |Sph|

‖=Absolute value

* Negative cylinder notation was used to apply this classification.

† Included myopia and myopic astigmatisms.

‡ Included hyperopia and hyperopic astigmatism.

SE, spherical equivalent.

### Data/Statistical analysis

The data were analysed and visualised via Stata Statistical Software: Release 18 (StataCorp LLC, College Station, TX, USA; https://www.stata.com). Diagnostic accuracy metrics (sensitivity, positive and negative predictive values, specificity, kappa, and area under the curve (AUC)) were calculated to evaluate the precision of the refraction measures obtained through the refractive procedures.

A receiver operating characteristic (ROC) analysis was conducted in accordance with the methodology proposed by DeLong et al.^21^ An AUC greater than 0.9 is considered excellent, 0.8 to -0.9 signifies very good accuracy, and 0.7 to -0.8 indicates good accuracy. An AUC between 0.6 and 0.7 signifies moderate accuracy, whereas an AUC below 0.6 denotes poor accuracy. The data were analysed in accordance with the methodology proposed by Thibos et al.^22^, in which astigmatism was decomposed into power vector notation (J_0_ and J_45_, where J_0_ denotes vertical/horizontal astigmatism and J_45_ denotes oblique astigmatism). Additionally, Bland–Altman analysis was employed to evaluate the agreement between retinoscopy and autorefraction and noncycloplegic subjective refraction measures of cylindrical errors and spherical equivalents. The 95% limits of agreement were determined as the means of differences with the standard deviations, and a p-value < 0.05 was considered statistically significant.

### Ethical considerations

Ethical approval was obtained from the Institutional Review Board (IRB) of the University of Cape Coast, Ghana, with reference number UCCIRB/CHAS/2024/020. Permission was sought from the Administration and the Head of the Optometry Department of Bishop Ackom Memorial Christian Eye Centre. The study's goal and methodology were explained to participants prior to obtaining written informed consent. This study adhered to the tenets of the Declaration of Helsinki for research on human studies.

## Results

One hundred young adults participated in this study (200 eyes). The majority of the participants (61, 61%) were female. The participants' ages ranged from 18 to 35 years (mean age of 23.38 ± 4.39 years). Descriptive statistics for power vector values obtained from retinoscopy, autorefraction and subjective refraction (segregated by refractive error type) are presented in Table 2.

**Table 2:** Descriptive statistics for power vector values obtained from retinoscopy, autorefraction and subjective refraction (segregated by refractive error type)

Refractive Technique		Emmetropia (n,%)	Hyperopia (n,%)	Myopia (n,%)	Total (n,%)
	N	79 (39.5%)	17 (8.5%)	104 (52.0%)	200 (100.0%)
**Retinoscopy**	M	-0.036 (0.321)	0.581 (0.955)	-1.962 (0.977)	-0.985 (1.293)
	J_0_	0.008 (0.253)	0.132 (0.323)	0.106 (0.538)	0.069 (0.431)
	J_45_	0.006 (0.026)	-0.000 (0.000)	0.006 (0.045)	0.006 (0.036)
**Autorefraction**	M	-0.252 (0.386)	0.331 (1.142)	-2.427 (1.117)	-1.333 (1.461)
	J_0_	0.197 (0.331)	0.277 (0.455)	0.323 (0.604)	0.269 (0.502)
	J_45_	0.134 (0.187)	0.153 (0.208)	0.029 (0.437)	0.081 (0.345)
**Subjective Refraction**	M	-0.014 (0.206)	0.846 (0.594)	-1.887 (0.938)	-0.915 (1.257)
	J_0_	0.047 (0.152)	0.088 (0.196)	0.141 (0.513)	0.099 (0.388)
	J_45_	-0.000 (0.000)	-0.000 (0.000)	-0.017 (0.170)	-0.009 (0.122)

### Comparison of refractive measurements between autorefraction, retinoscopy and subjective refraction

This study assessed agreement between spherical equivalents and cylindrical components of autorefraction, retinoscopy, and subjective refraction using Bland–Altman analyses and intraclass correlation coefficients (ICCs). Bland–Altman plots comparing measurements between refractive techniques are shown in Figure 1a-f. For spherical equivalents, retinoscopy and subjective refraction demonstrated strong agreement (ICC=0.9), whereas autorefraction and subjective refraction also showed significant agreement (ICC=0.8, p < 0.001), with a mean bias of -0.418D ± 0.45 and limits from -1.3110D to 0.4748D (Figure 1a-b). For the Jackson cross-cylinder component along the 0° axis (J_0_), retinoscopy and subjective refraction were significantly correlated (ICC=0.81, p < 0.001), with a mean bias of -0.30 ± 0.25 and limits from -0.5292 to 0.4687; similarly, autorefraction and subjective refraction for J_0_ were correlated (ICC=0.67, p < 0.001), with a mean bias of 0.17 ± 0.32 and limits from -0.4759 to 0.8161 (figure 1c-d). Additionally, for the J_45_ axis, significant but low agreement was noted between retinoscopy and subjective refraction (ICC=0.01, p=0.48), with a mean bias of 0.014 ± 0.12 and limits from -0.2353 to 0.2638, whereas autorefraction and subjective refraction on the J_45_ axis demonstrated a weak but significant correlation (ICC=0.22, p < 0.001), with a mean bias of 0.089 ± 0.314 and limits from 0.5264 to 0.7059 (figure 1c-d).

**Figure 1a:**
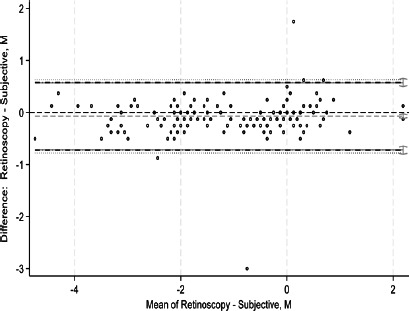
Bland-Altman plots comparing refractive measurements between retinoscopy and subjective refraction (spherical equivalent)

**Figure 1b:**
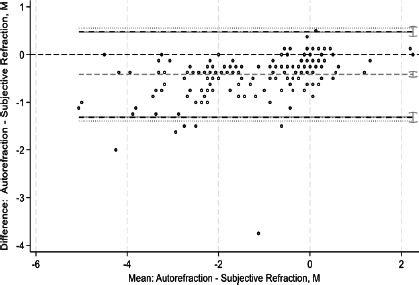
Bland-Altman plots comparing refractive measurements between auto refraction and subjective refraction (spherical equivalent)

**Figure 1c:**
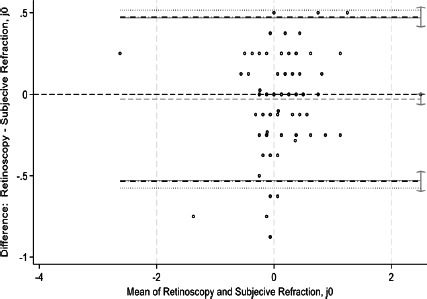
Bland-Altman plots comparing refractive measurements between retinoscopy and subjective refraction (J_0_ AXIS)

**Figure 1d:**
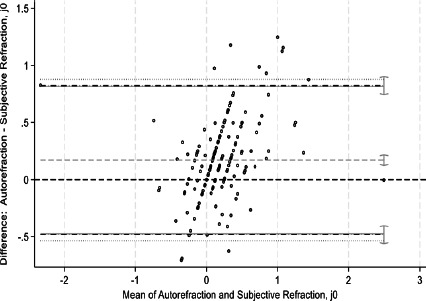
Bland-Altman plots comparing refractive measurements between Auto refraction and subjective refraction (J_0_ AXIS)

**Figure 1e:**
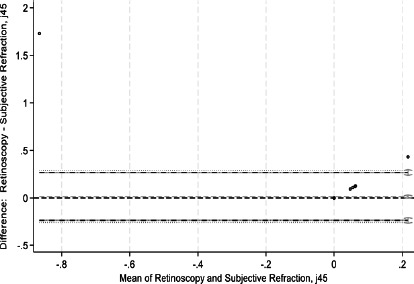
Bland-Altman plots comparing refractive measurements between retinoscopy and subjective refraction (J_45_ AXIS)

**Figure 1f:**
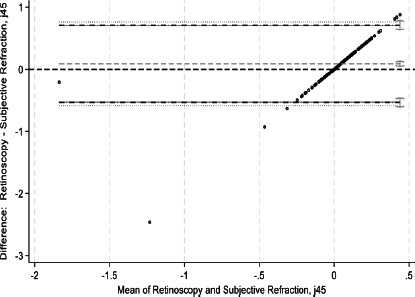
Bland-Altman plots comparing refractive measurements between Auto refraction and subjective refraction (J_45_AXIS)

Figure 1 shows Bland–Altman plots comparing refractive values between a. retinoscopy vs subjective refraction (spherical equivalent); b. autorefraction vs subjective refraction (spherical equivalent); c. retinoscopy vs subjective refraction (J_0_); d. autorefraction vs subjective refraction (J_0_); e. retinoscopy vs subjective refraction (J_45_); and f. autorefraction vs subjective refraction (J_45_).

### Correlations between refractive values and autorefraction, retinoscopy and subjective refraction

**Figure 2(a-f):**
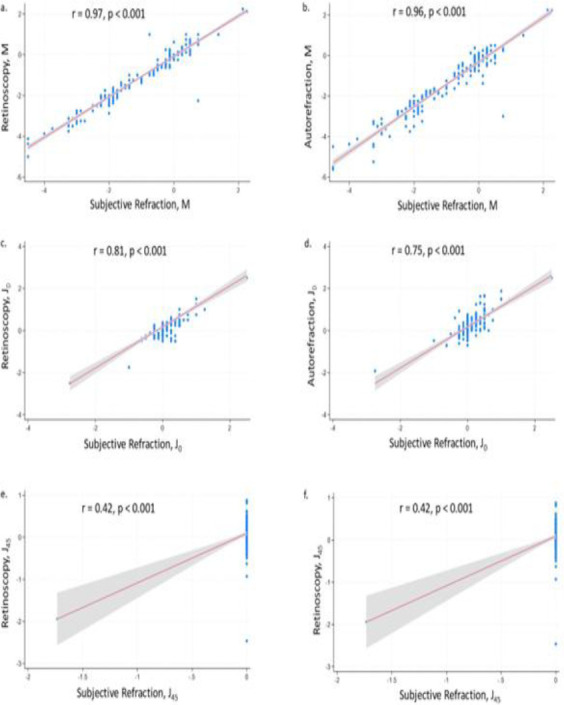
Correlations between retinoscopy, autorefraction, and subjective refraction

The figures (2a-b) show strong correlations between subjective refraction and both retinoscopy (r = 0.97) and autorefraction (r = 0.96) for spherical measurements (M), suggesting high agreement between these methods for determining spherical power. Moderate correlations appear in the J_0_ measurements between retinoscopy and subjective refraction (r = 0.81) and autorefraction (r = 0.75), indicating fair agreement for this component (2c-d). However, there was a near-zero correlation in J_45_ between subjective refraction and retinoscopy (r = 0.01, nonsignificant) and only a weak correlation with autorefraction (r = 0.42), suggesting poor alignment for astigmatic measurements in this axis (2e-f).

### Sensitivity and specificity of retinoscopy and autorefraction in the detection of refractive errors

**Figure 3:**
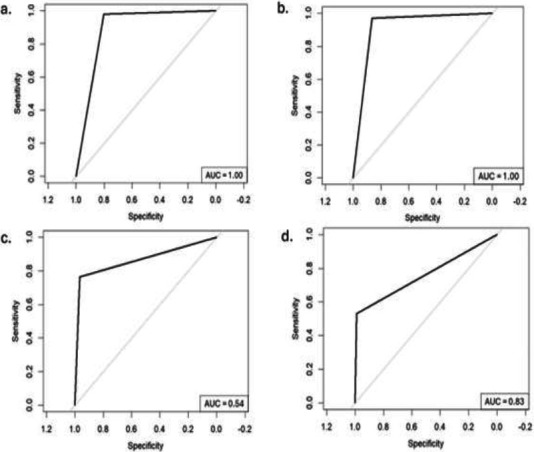
ROC curves for the detection of myopia and hyperopia by retinoscopy and autorefraction (a. detection of myopia by retinoscopy; b. detection of myopia by autorefraction; c. detection of hyperopia by retinoscopy; d. detection of hyperopia by autorefraction)

Receiver operating characteristic (ROC) curves were used to assess the diagnostic performance of retinoscopy and autorefraction in detecting myopia and hyperopia, using subjective refraction as the reference standard (Figure 3). The Youden index^23^ was used to ascertain the retinoscopy and autorefractor thresholds that maximised specificity and sensitivity for each ROC curve. The sensitivity and specificity of retinoscopy in detecting myopia were similar to those of autorefraction at the optimum threshold (Table 3). For myopia detection, the AUC results were excellent for both retinoscopy (AUC = 1.00) and autorefraction (AUC = 1.00) (Figure 3).

**Table 3:** Coefficient indices for retinoscopy and autorefraction in the detection of refractive errors

	Myopia		Hyperopia	

	Retinoscopy	Autorefraction	Retinoscopy	Autorefraction
**Accuracy (%)**	92.00	89.5	95.00	95.00
	[87.33 to 95.36]*	[84.4 to 93.38]*	[91.00 to 97.58]*	[91.00 to 97.58]*
**Sensitivity (%)**	86.46	80.21	96.72	98.91
**Specificity (%)**	97.12	98.08	76.47	52.94
**PPV (%)**	96.51	97.47	97.79	95.77
**NPV (%)**	88.60	84.30	68.42	81.82
**Kappa (%)**	83.91	78.82	69.48	61.73

PPV – Positive Predictive value

NPV – Negative Predictive Value

* 95% Confidence Interval

Computed threshold was 0.5 for each analysis, based on Youden's model

The accuracy of retinoscopy was greater than that of autorefraction in detecting myopia. However, retinoscopy and autorefraction demonstrated equal accuracy in detecting hyperopia (Table 3). Kappa was greater in the detection of myopia by both methods of refraction than in the detection of hyperopia (Table 3).

## Discussion

This study sought to assess, in young adults, the agreement among retinoscopy, autorefraction, and subjective refraction in assessing refractive errors, specifically, the spherical equivalent, J_0_, and J_45_ axes, via the intraclass correlation coefficient (ICC), Bland–Altman analysis and receiver operating characteristics. For spherical equivalents, our overall results revealed a strong degree of agreement between retinoscopy and subjective refraction, whereas retinoscopy was more precise than autorefraction was, which is consistent with the findings of similar studies.^24^–^26^ These differences may be attributed to the inherent limitations of autorefraction, fixation instability, and instrument myopia, especially in younger adults with active accommodative responses. Retinoscopy, by contrast, allows real-time control of working distance and accommodation, enabling the examiner to neutralise reflexes more accurately.

Although retinoscopy exhibited smaller limits of agreement, suggesting greater consistency, both retinoscopy (ICC = 0.81, p < 0.001) and autorefraction (ICC = 0.67, p < 0.001) showed strong agreement with subjective refraction for the J_0_ component. The two techniques, however, showed significant but weak agreement with subjective refraction for the J_45_ axis. These findings imply that although retinoscopy and autorefraction are accurate for measuring spherical equivalents and J_0_, they may not be precise for J_45_ estimation due to their limited reliability. This limited dependability likely reflects the difficulty of accurately detecting oblique astigmatism, as J_45_ is more sensitive to small alignment errors, fixation instability, and residual accommodation than spherical equivalents or J_0_.

These results match earlier studies^26^–^28^, which noted a stronger alignment between retinoscopy and subjective refraction than between retinoscopy and autorefraction.

These findings suggest that clinicians should consider retinoscopy before performing autorefraction. Additionally, with contemporary advances in artificial intelligence (AI), more precise autorefraction and AI-assisted retinoscopy equipment will be on the market.^26^ These advancements could improve the accuracy of autorefraction results and ultimately enhance patient care. It is important for clinicians to keep up with technological developments to provide the best possible care for their patients.

In terms of correlation analysis, there was strong agreement between subjective refraction and both retinoscopy and autorefraction for spherical power (M), as evidenced by high correlation coefficients (r = 0.97 and r = 0.96, respectively). This suggests that either retinoscopy or autorefraction can be reliably used alongside subjective refraction to assess spherical refractive errors, yielding consistent measurements across methods. In the case of astigmatism, however, the reliability diminishes, particularly for the J_45_ component, where retinoscopy showed no significant correlation (r = 0.01), and autorefraction demonstrated only weak alignment (r = 0.42). This finding indicates that while spherical power can be measured accurately across different refraction methods, astigmatic measurements, especially along the J45 axis, may require caution, as variability between methods could affect clinical interpretation, a finding similar to Blant–Altman–s analysis.

Using Youden's model, the results for predicting myopia and hyperopia with retinoscopy and autorefraction show distinct levels of sensitivity, specificity, and accuracy across the refractive error types. For myopia prediction, retinoscopy achieved a sensitivity of 0.97, a specificity of 0.86, and an accuracy of 0.92, suggesting high reliability in detecting true myopia cases while maintaining substantial specificity. Compared with retinoscopy, autorefraction performed similarly, with slightly higher sensitivity (0.98), lower specificity (0.80), and accuracy (0.90), indicating strong sensitivity but a tendency toward more false positives. For hyperopia prediction, retinoscopy demonstrated a sensitivity of 0.77, specificity of 0.97, and accuracy of 0.95, making it effective for confirming hyperopic cases with high specificity, although with slightly lower sensitivity. However, autorefraction showed a sensitivity of only 0.53, with a high specificity of 0.99 and an accuracy of 0.95, indicating that it is highly specific but less sensitive, potentially under detecting true hyperopia cases. Overall, both methods show high accuracy in hyperopia prediction, with retinoscopy being more balanced across sensitivity and specificity, whereas autorefraction exhibits strong specificity but lower sensitivity.

The efficiency of autorefraction makes it especially appropriate for high-volume screenings^26^,^29^ The accuracy of retinoscopy may be more helpful in settings where thorough refractive examinations are needed.^26^ In support of our conclusions and implying that autorefraction can be useful for cylindrical power assessment when subjective refraction is not practical, the spherical component, as reported, often exhibits greater agreement with autorefraction.^26^,^29^ These results match earlier studies^24^–^26^, which noted a stronger alignment between retinoscopy and subjective refraction for the spherical component, with autorefraction tending to somewhat overestimate myopia and overestimate hyperopia.^25^ Additionally, Asiedu et al.,^19^ suggested retinoscopy for overall astigmatism assessments even though autorefraction offers a useful starting point for subjective refraction of the cylindrical component.

This study has several limitations that may affect the generalizability of the findings. The pertinent one was the unequal presentation of the types of refractive errors. Future research should aim to include a more balanced representation of refractive errors to improve the overall validity and applicability of the findings. Additionally, a larger, more diverse sample could help address this limitation and provide a more comprehensive understanding of the topic. The study used a closed-field Humphrey Zeiss autorefractor without cycloplegia, which may have induced instrument myopia (due to proximal accommodation) and biased autorefraction toward more myopic values or underestimation of hyperopia.

Although participants were instructed to fixate on the distant target and alignment was carefully controlled, residual accommodation cannot be ruled out and may partly explain the wider limits of agreement observed in autorefraction. In addition, the unequal distribution of refractive error types and the modest sample size limit generalizability. Future studies should consider using an open-field autorefractor or applying cycloplegia in a sub-sample to quantify and control for accommodative effects, and should aim for a larger, more balanced sample across refractive error categories.

## Conclusion

In good agreement with subjective refraction in a sample of young people, this study shows retinoscopy's consistency in evaluating spherical equivalents and the J_0_ axis. Although efficient, autorefraction showed reduced precision and less consistency for J_45_ axis determination. Particularly for young adult populations, these results can guide clinicians in selecting appropriate refractive techniques based on patient needs and the clinical setting.
